# Human infections due to *Mycobacterium lentiflavum*: first report in Iran

**Published:** 2010-03

**Authors:** M Shamaei, M Marjani, P Farnia, P Tabarsi, D Mansouri

**Affiliations:** 1 Clinical Tuberculosis and Epidemiology Research Center; 2 Mycobacteriology Research Center; 3 Chronic Respiratory Disease Research Center, NRITLD, Masih Daneshvari Hospital, Shahid Beheshti University of Medical.Sciences, Tehran, Iran

**Keywords:** *Mycobacterium lentiflavum*, NTM, Iran

## Abstract

Non-tuberculosis mycobacteria (NTM), as certain species of mycobacteria, can affect human in several ways. In the preceding years, the rate of NTM detection has risen in both immunocompromised and immunocompetent patients. On the other hand, several reports have debated the possibility of co-infection of both *Mycobcateriu tuberculosis* (MTB) and NTM in individuals that puts the role of NTM in disease manifestations under question. Moreover, it is now proven that some of the cases that are identified as anti-TB treatment failure or suspected for drug resistance are actually NTM.

## INTRODUCTION

Non-tuberculous mycobacteria, as certain species of mycobacteria, can affect humans in several ways (1–3). Though they are thought to affect the immunocompromised patients more frequently, several studies have demonstrated NTM as the etiological agent of disease in immunocompetent hosts (4–6).

In the past few years, rate of NTM detection has risen in both immunocompromised and immunocompetent patients. This may be partly attributed to advances in mycobacteriologic detection methods, increase in acquired immunodeficiency cases (either HIV infection or use of immunosuppressive drugs), or just simply actual increase in NTM infection itself (1).

On the other hand, several reports have debated the possibility of co-infection of both MTB and NTM in an individual that puts the role of NTM in disease manifestations under question. Moreover, it has now been proven that some cases that are identified as anti- TB treatment failure or suspected for drug resistance are actually NTM (1).

A broad spectrum of non-tuberculous mycobacteria live freely in our surrounding environment (1–3). However, they may cause disease in both human or animals. Human to human transmission of NTM has not yet been confirmed. Though, animal to human transmission has been suggested in some reports (7, 8). So, role of NTM, particulalry when public health issues are concerned, should be emphasized despite the fact that no definitive route of transmission is yet proposed (i.e., either human to human or animal to human).

NTM and the diseases they cause have received much attentions world-wide during the past decade. Also, with regard to the enormity of varieties of these organisms, the sporadic diseases caused by them, and the difficulties in detecting such bacilli, the reported cases are still somewhat few. Therefore, any kind of disease caused by NTM, especially those presenting with clinical manifestations during their involvement and rare cases would merit being reported for further considerations.

*Mycobacterium lentiflavum* was first identified in 1996 as a disticnt strain (9). Since then, several cases of infections with this bacilli have been reported with skin or lymph node involvement. Also, it has been isolated from pleural effusions, ascites, and from lung tissues (10–12).

This paper is the first report of documented *M. lentiflavum* infection from Iran.

## Case Report

A 44 year-old Iranian woman who was resident of Kurdestan province in the west of Iran, presented to our center with cough, scanty sputum, left unilateral pleuritic chest pain, anorexia and weight loss for the last three months that had exacerbated in the preceding couple weeks.

In 1999, her symptoms first started and thus she underwent an assessement for TB. The clinical manifestations typical for and the radiological findings consistent with TB, accompanied with positive sputum smear for Acid Fast Bacilli, led to the initiation of anti-TB treatment. She had received the WHO CAT ? regimen for six months at a peripheral health care center in Iran. Since then, the patient has been treated with anti-TB medication three times, including CAT ?? regimen, due to recurrence of clinical manifestations and each time the treatment course was completed and cure was documented by obtaining negative sputum smear negative for AFB.

For the last three months, and the first time after her third treatment regimen, her symptoms re-emerged. She was evaluated at the peripheral health care center and due to a positive smear for AFB, was referred to our center as a suspected case for MDR-TB.

At admission, she appeared quite cachectic. There was no cervical or axillary lymphadenopathy. In chest auscultation, inspiratory crackles were detected mainly on the left middle and lower regions. Tuberculin skin test (TST) performed with PPD revealed 10 mm of induration. No BCG vaccination scar was detected.

As a suspected MDR-TB case and according to our routine protocol for these patients, mycobacteriologic studies (including sputum smears and cultures, and Drug Suscpetibility Testing (DST)), polymerase chain reaction (PCR) specific for MTB, and other identification tests were requested. The sputum smears rendered a 2+ result.

The patient, as a suspected MDR-TB case, underwent the standardized anti-TB regimen consisting of cycloserine, ofloxacin, prothionamide, and amikacin.

**Fig. 1:**
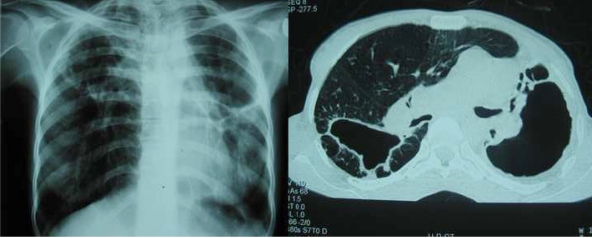
Chest Radiography, PA, and Conventional CT Scan on July 2007, Demonstrate bilateral fibrocavitary destructive changes and biapical pleural thickening.

The anti-HIV antibody test was negative. Sputum smear was 2+ for AFB. Chest X-ray showed bilateral fibrocavitary destructive changes and biapical pleural thickening Chest CT scan revealed fibrocavitary destructive change in both upper lobes, heart and mediastane shift toward left side, biapical pleural thickening and loss of volume seen with compensatory over aerated and emphysematous lower lobe changes (Fig. 1).

Drug suscpetibility testing (DST) demonstrated resistance to all first line anti-TB drugs. PCR for MTB was negative and with further evaluations using identification methods, *Mycobcaterium lentiflavum* was identified in the specimens.

As *Mycobacterium lentiflavum* had been isolated, the treatment regimen changed to clarithromycin, Ofloxacin, and amikacin. The sputum culture turned negative after two months of treatment and has remained so for the time being while the patient has received more than six months medication.

Specimens obtained from the patient were cultured on the Lowenstein – Jensen (L.J.) slants so that 100–120 colonies grew. Whenever polymerase chain– reaction using RFLP-IS6110 probe and spoligotyping were negative, conventional biochemical tests as identification methods (13) were performed for both the patient strain as well as standard NTM (obtained from Department of Mycobacteriology, National Institute for Public Health and the Environment, the Netherlands). The tests included photo-induction test, niacin production, nitrate reduction, catalase (heat stable & Semiquantitative), tween hydrolysis (10 days), and urease (Murphy – Hawkins disk method). In addition, the growth on L.J. culture media at 25, 37, 40 and 45°C were determined.On the basis of data reported in Bergey's Manual of Systematic Bacteriology (13, 14), the only species matching the specific pattern appeared to be NTM.

## DISCUSSION

NTM most commonly presents with pulomonary manifestations. However, lymph node, skin, soft tissue involvement as well as disseminated disease are of clinical importance (1). *Mycobacterium lentiflavum* was first identified in 1996 as a distinct strain. Since then, several cases of infections with this bacilli have been reported with skin or lymph node involvements. Also, it has been isolated from pleural effusions, ascites, and from lung tissue.

However, the pulmonary disease caused by *M. lentiflavum* are few. Furthermore, most of the reported cases were detected in immunocompromised patients. It is of note that this case occurred in an immunocompetent patient and there are few such reports available (15) As well, after administering the approprite treatment for *M. lentiflavum*, the clinical condition of the patient improved, her sputum smears and culture turned negative; therefore, this is among few cases that the *M. lentiflavum* treatment outcome is determined.

The notable charcateristics of our case was that she had the history of several anti-TB treatments. According to NTP, anti-TB treatment is initiated based on positive smears, culture and DST are not routinely performed. So, we do not know about the type of mycobacterium in her previous illnesses.

Accordingly, in patients who are treated according to NTP (merely based on positive smears), NTM should be kept in mind especially if the patient does not properly respond to the standard anti-TB regimens. Moreover, despite some recommendations for treatment of *M. lentiflavum*, it seems that further evaluations should be developed to find the optimal therapeutic protocol and regimen.
